# Cathepsin V regulates cell cycle progression and histone stability in the nucleus of breast cancer cells

**DOI:** 10.3389/fphar.2023.1271435

**Published:** 2023-11-06

**Authors:** Naphannop Sereesongsaeng, James F. Burrows, Christopher J. Scott, Klaudia Brix, Roberta E. Burden

**Affiliations:** ^1^ School of Pharmacy, Medical Biology Centre, Queen’s University Belfast, Belfast, United Kingdom; ^2^ The Patrick G Johnston Centre for Cancer Research, Medical Biology Centre, Queen’s University Belfast, Belfast, United Kingdom; ^3^ School of Science, Constructor University, Bremen, Germany

**Keywords:** cysteine cathepsin, protease, histone, chaperone, nucleus, importin, breast cancer

## Abstract

**Introduction:** We previously identified that Cathepsin V (CTSV) expression is associated with poor prognosis in ER+ breast cancer, particularly within the Luminal A subtype. Examination of the molecular role of the protease within Luminal A tumours, revealed that CTSV promotes tumour cell invasion and proliferation, in addition to degradation of the luminal transcription factor, GATA3, via the proteasome.

**Methods:** Cell line models expressing CTSV shRNA or transfected to overexpress CTSV were used to examine the impact of CTSV on cell proliferation by MTT assay and flow cytometry. Western blotting analysis was used to identify the impact of CTSV on histone and chaperone protein expression. Cell fractionation and confocal microscopy was used to illustrate the presence of CTSV in the nuclear compartment.

**Results:** In this work we have identified that CTSV has an impact on breast cancer cell proliferation, with CTSV depleted cells exhibiting delayed progression through the G2/M phase of the cell cycle. Further investigation has revealed that CTSV can control nuclear expression levels of histones H3 and H4 via regulating protein expression of their chaperone sNASP. We have discovered that CTSV is localised to the nuclear compartment in breast tumour cells, mediated by a bipartite nuclear localisation signal (NLS) within the CTSV sequence and that nuclear CTSV is required for cell cycle progression and histone stability in breast tumour cells.

**Discussion:** Collectively these findings support the hypothesis that targeting CTSV may have utility as a novel therapeutic target in ER+ breast cancer by impairing cell cycle progression via manipulating histone stabilisation.

## Introduction

In recent years, increasing evidence supports the involvement of the cysteine cathepsin proteases in a wide range of physiological and pathological processes, ranging from antigen presentation and wound healing to tumour cell invasion and inflammatory pain (Kramer et al., 2017; Yadati et al., 2020; Biasizzo et al., 2022). In the context of malignancies, there has been a focus on the molecular mechanisms by which Cathepsin B, L and S contribute to disease, however there is now increasing evidence to support the involvement of some of the lesser studied family members, in particular Cathepsin V (CTSV) (reviewed by Lecaille et al., 2022).

Elevated CTSV expression has been observed in numerous cancer types, including squamous cell, breast, colorectal and thymic epithelial malignancies (Santamaria et al., 1998; Haider et al., 2006; Sun et al., 2016; Kiuchi et al., 2017), and associated with increasing tumour grade/stage in hepatocellular and endometrial malignancies (Skrzypczak et al., 2012; Jing et al., 2018). CTSV has been identified as a highly potent elastase (Yasuda et al., 2004), whose effect is mediated via the presence of two hydrophobic exosites that stabilize CTSV and elastin complex formation. The presence of these exosites on CTSV explains why highly homologous CTSL (78% homology to CTSV) has such weak elastinolytic activity in comparison, as replacement of the exosite sequences within CTSV significantly impairs elastase activity (Du et al., 2013).

From a mechanistic perspective, there have been multiple pro-tumourigenic behaviours associated with CTSV. In the context of endometrial carcinoma, CTSV expression has been shown to be positively correlated with several proteins involved in the regulation of cell cycle and proliferation, including MYB proto-oncogene like 2 (MYBL2), p21/WAF, cyclin B1, Ki-67 and human epidermal growth factor receptor 2 (HER2) (Skrzypczak et al., 2012). Wang and others identified an inverse correlation between CTSV mRNA expression and tumour suppressor tazarotene-induced gene 1 (TIG1) in colon cancer. They also determined that CTSV promotes uPA activity and epithelial-to-mesenchymal transition (EMT) within HCT116 cells, both of which contribute to tumourigenesis. Interestingly, they discovered that TIG1 overexpression can also inhibit the expression of CTSV, suppressing CTSV-induced invasion and migration (Wang et al., 2020). Whether these correlations extend to other cancer types, remains to be determined but it does suggest that the examination of CTSV in malignancy warrants further investigation.

We have previously reported that elevated CTSV expression is associated with a poor clinical outcome in ER+ (Luminal A) breast cancer patients (Sereesongsaeng et al., 2020). We identified that CTSV regulates protein expression of the luminal transcription factor, GATA3, promoting its degradation via the proteosome. Collectively, this work suggested that CTSV may represent an exciting new therapeutic target in ER+ breast cancer, however further work needed to be done to understand the molecular intricacies of CTSV function. In this study we have identified that CTSV depletion attenuates tumour cell proliferation, by mediating a delay in cell cycle progression. We have identified that CTSV has a role in regulating histone H3 and H4 protein levels by controlling expression of the histone chaperone, sNASP. Intriguingly, our findings suggest that this is mediated by CTSV localised to the nuclear compartment via a predicted bipartite nuclear localisation sequence and importin trafficking. Collectively, these results suggest that CTSV is localised to the nuclear compartment in breast cancer cells and that targeting CTSV may have potential application to reduce tumour growth by impairing cell cycle progression for ER+ breast cancer.

## Materials and methods

### Cell line culture and treatments

Human ER+ breast cancer cell lines MCF-7 and ZR75-1 were acquired from the American Type Culture Collection (ATCC). MCF-7 cells were cultured in Dulbecco’s Modified Eagle Medium (DMEM) supplemented with 10% foetal calf serum (FCS), 50 U/mL penicillin and 50 μg/mL streptomycin. ZR75-1 cells were cultured in Roswell Park Memorial Institute-1640 media (RPMI-1640) supplemented with 10% foetal calf serum, 1% sodium pyruvate, 50 U/mL penicillin and 50 μg/mL streptomycin. Cell treatments with ivermectin (10 μM, Sigma-Aldrich) were undertaken for 24 h. All cells were cultured at 37 °C in a humidified incubator with 5% CO_2_. All cell culture media, supplements and antibiotics were purchased from Thermo Fisher Scientific.

### Lentiviral cell line generation and transient transfections

Lentiviral cell line generation was undertaken and validated as previously described (Sereesongsaeng et al., 2020). Briefly, 293T cells were transfected with pLKO.1-shCTSV (sh1, sh2) and non-targeting control (NTC) plasmids (Sigma-Aldrich), after which viral supernatant was harvested and used to transduce MCF-7 and ZR75-1 cells. For transient transfections, cells were transfected using GeneJuice^®^ transfection reagent (Merck Millipore) in accordance to manufacturer’s instructions. Cells were transfected with 2 μg of DNA and incubated for 48 h prior to downstream analysis. Plasmids utilised for transient transfections were all derived from pcDNA3.1-CTSV ORF (GeneScript), with pcDNA3.1 used as an empty vector control.

### Cell viability

Cells were seeded at the appropriate density in complete growth media in 96-well plates with a final volume of 200 μL and incubated at 37 °C in a humidified incubator at 5% CO_2_ overnight. MTT solution was prepared from 5 mg/mL MTT (Sigma-Aldrich) in sterilised PBS which was filtered and stored in the dark at 4 °C. To each well, 10 μL of MTT solution was added to the growth media and plates were incubated at 37 °C for 3 h. Following incubation, MTT solution was removed and 50 μL of DMSO was added to each well to dissolve the formazan crystals. Plates were shaken for 10 min to ensure adequate solubilisation and absorbance readings from each well were measured at 570 nm using a Cytation™ 5 plate reader and Gen5 software (BioTek). All assays were performed with five sample replicates and repeated at least three independent times. The average and standard deviation (SD) values were collated from three independent experiments and statistical analysis was determined by one-way-ANOVA using GraphPad Prism 8.

### Confocal microscopy

Cells were seeded in 8 chamber glass culture slides (BD Falcon) for 24 h before experimentation. Fixation was carried by incubating with 4% paraformaldehyde for 30 min in the dark, with cell permeabilization undertaken using 0.1% Triton X-100 in PBS for 10 min at room temperature. After blocking with 1% BSA and 10% donkey serum in PBS for 1 h at 37 °C, cells were incubated with a CTSV primary antibody (BioTechne, AF1080), prepared in blocking solution and incubated at 4 °C for 24 h. Alexa Fluor 568 donkey anti-goat (A11057, Thermo Fisher Scientific), used as a secondary antibody, was incubated for 1 h at 37°C and cell nuclei were counter-stained using ProLong™ Glass Antifade Mounting media (Thermo Fisher Scientific). As a control, incubation with only the secondary antibody was also performed. A Leica SP8 confocal Microscope was used to view and analyse images, with a minimum of 15 fields of view captured per well. Fluorescent images were captured with ×63 oil immersion objective lens and mages were analysed with Fiji ImageJ software version 2.0.

### Cell synchronisation

In order to synchronise the MCF-7 cells, cells were treated with 2.5 mM thymidine (Sigma-Aldrich) for 16 h, followed by release for 8 h. Cells were then incubated with 2.5 mM thymidine for a further 16 h to arrest cells at G1 phase. Cells were released and collected at 4 h and 8 h to obtain S and G2/M phases, respectively. For the ZR75-1 cells, cells were sequestered in G1 phase by arresting cells with 2.5 mM thymidine for 18 h, followed by releasing cells for 9 h and arresting cells with 2.5 mM thymidine for a further 17 h. To obtain cell populations in S phase, cells were arrested with 2.5 mM thymidine for 18 h and released for 4 h before collecting cells. To obtain cell populations in G2/M phase, cells were arrested with 100 ng/mL nocodazole (Sigma-Aldrich) for 16 h.

### Flow cytometry

Cells were detached using 1% w/v EDTA/PBS, incubated for 5 min and collected by centrifugation at 300 *g* for 3 min. Cell pellets were initially resuspended with 5% FCS/PBS before centrifuging at 850 g for 3 min, with cell fixed in 70% ethanol at 4 °C overnight. After complete fixation, the cells were washed with PBS before proceeding with cell staining using FxCycle™ PI/RNase Staining Solution (Thermo Fisher Scientific). All samples were incubated for 15 min at 37 °C, while protected from the light. Cell cycle phase distributions were subsequently analysed by flow cytometry using a FACS Calibur (BD Bioscience).

### Western blotting

Cell pellets were harvested by centrifugation before lysis in RIPA buffer containing the mini cOmplete™ protease inhibitor cocktail (Roche). The cell suspension was incubated on ice for 30 min with intermittent vortexing at 5 min intervals. Following this, the suspension was centrifuged at 15,000 g for 10 min at 4 °C and extracted proteins from the supernatant was collected. All protein quantification was undertaken using the Pierce™ BCA Protein assay kit (Thermo Fisher Scientific). Protein lysates were resolved by SDS-PAGE using 10% acrylamide gels, and following electrophoresis, proteins were transferred onto polyvinylidene fluoride (PVDF) membranes by semi-dry blotting. The following antibodies were used in this study; goat polyclonal CTSV (BioTechne, AF1080), goat polyclonal cyclin D1/D2 (AF4196, BioTechne), mouse monoclonal cyclin E1 (MAB68101, BioTechne), rabbit monoclonal cyclin B1 (MAB60001, BioTechne), rabbit polyclonal HDAC1 (2062S, Cell Signaling), mouse monoclonal histone H1 (NBP2-45184, BioTechne), rabbit polyclonal histone H2a (NB100-56346, BioTechne), rabbit polyclonal histone H2b (NB100-56633, BioTechne), rabbit monoclonal histone H3 (4499S, Cell Signaling), rabbit monoclonal histone H4 (NBP2-80444, BioTechne), mouse monoclonal Hsc70 (MAB4148, BioTechne), mouse monoclonal Hsp90 (ab13492, Abcam), mouse monoclonal Asf1b (NBP2-61684, BioTechne), rabbit monoclonal NASP (ab181169, Abcam), mouse monoclonal GATA3 (BioTechne, MAB6330), goat polyclonal GAPDH (BioTechne, AF5718), mouse monoclonal CTSL (BioTechne, MAB9521) and rat monoclonal α-Tubulin (ab6160, Abcam). Secondary antibodies used were donkey anti-goat HRP conjugate (HAF109, BioTechne), goat anti-mouse HRP conjugate (172–1011, BioRad), goat anti-rabbit HRP conjugate (7074, Cell Signaling) and goat anti-rat HRP conjugate (ab205720, Abcam). Protein visualisation was undertaken using the Luminata™ Forte chemiluminescent detection reagent (Merck Millipore) and imaged using a ChemiDoc XRS + Imaging System (BioRad). Densitometry analysis was performed using the Image Lab software on the ChemiDoc XRS + Imaging System (BioRad), with at least 3 independent experiments. Results are presented as mean relative densitometry units and standard deviation (SD) values, with relative expression compared to control samples, and with the adjusted intensity (subtraction of the background signal) used for all calculations.

### Nuclear and cytoplasmic extractions

Cells were harvested by incubating with 1% EDTA for 5 min at 37 °C, followed by washing with ice-cold PBS. Cell pellets were resuspended in hypotonic buffer (20 mM Tris-HCl, 10 mM NaCl, 3 mM MgCl_2_), incubated on ice, and then vortexed with 10% NP40 prior to centrifugation. The supernatant, representative of the cytoplasmic fraction was collected. Cell pellets were resuspended in Cell Lysis Buffer (Thermo Fisher Scientific) containing 1 mM PMSF and cOmplete™ protease inhibitor cocktail (Roche) and incubated on ice, with vortexing at 10 min intervals. After centrifugation, the supernatant comprising the nuclear proteins was collected. Uncropped western blot images are presented in Supplementary Figures S1–S6.

### Site-directed mutagenesis

pcDNA3.1-CTSV ORF (GeneScript) was used as the DNA template for all constructs utilised within this work. The shRNA resistant plasmid (rCTSV) was generated by site-directed mutagenesis of the shCTSV-1 target sequence to alter codon usage but generate the same protein sequence. The NLS mutant construct (mtNLS) was also generated via site-directed mutagenesis, with arginine and lysine residues within the predicted bipartite NLS motif being mutated to alanine residues. All site-directed mutagenesis was undertaken using the QuikChange Lightning kit (Agilent) according to manufacturer’s instructions, using primers synthesised by MWG Eurofins. Successful mutagenesis was confirmed by DNA sequencing using the Genomics Core Technology Unit within QUB.

### RQ-PCR

RNA was extracted using Stat-60 (Amsbio) and cDNA was prepared using the Transcriptor First Strand cDNA synthesis kit (Roche) according to manufacturer’s instructions. RQ-PCR was undertaken using the Lightcycler^®^ 480 SYBR Green I Master reagent (Roche). Primer sequences used were as follows; CTSV-F: 5′-gga​ctc​tga​gga​atc​cta​tcc​at-3′, CTSV-R: 5′-gca​aca​gaa​ttc​tca​ggt​ctg​tac​t-3′, HIST1H3-F 5′-cgg​att​acc​aga​aat​cag​gag​t-3′, HIST1H3-R 5′- ttt​cgt​aga​gac​ggg​att​cg-3′, HIST1H4-F 5′-ggt​ccc​acc​ctc​atc​tcc-3′, HIST1H4-R 5′- gcc​tac​agt​ccg​ctc​ctt​ta-3′, sNASP-F 5′-cat​gga​gtc​cac​agc​cac​t-3′, sNASP-R 5′- cac​cat​tct​cca​ttc​tct​cca-3′, β-tubulin-F: 5′-cgc​aga​aga​gga​gga​gga​tt-3′, β-tubulin-R: 5′-gag​gaa​agg​ggc​agt​tga​gt-3′. RQ-PCR was performed in duplicate for each gene investigated, with all experiments undertaken a minimum of 3 times. The fold change of mRNA expression was calculated by normalising absolute target gene expression levels to β-tubulin mRNA levels as an internal control. The average and standard deviation (SD) values were collated from three independent experiments and statistical analysis was determined by one-way-ANOVA using GraphPad Prism 8.

### *In vitro* cleavage assay

Recombinant CTSV (1080-CY-010, BioTechne) and CTSL (952-CY-010, BioTechne) were activated before analysis according to manufacturer’s instructions. Activated CTSV or CTSL (8 ng) were incubated with 4 μg recombinant Histone H3 (rhH3) (ab198757, Abcam) and added to the recommended assay buffer (pH 5.5) to a final volume of 15 μL. Samples were incubated at 37 °C, with samples collected at defined timepoints. Samples were denatured at 95 °C for 10 min with Laemmli buffer before loading onto 15% acrylamide SDS-PAGE gels. rhH3, active CTSV and active CTSL were used as negative controls. The gels were subsequently washed with dH2O and stained with GelCode™ Blue Stain Reagent (Thermo Fisher Scientific) according to manufacturer’s instructions.

## Results

### CTSV depletion impedes breast cancer growth via a delay in cell cycle progression

**FIGURE 1:**
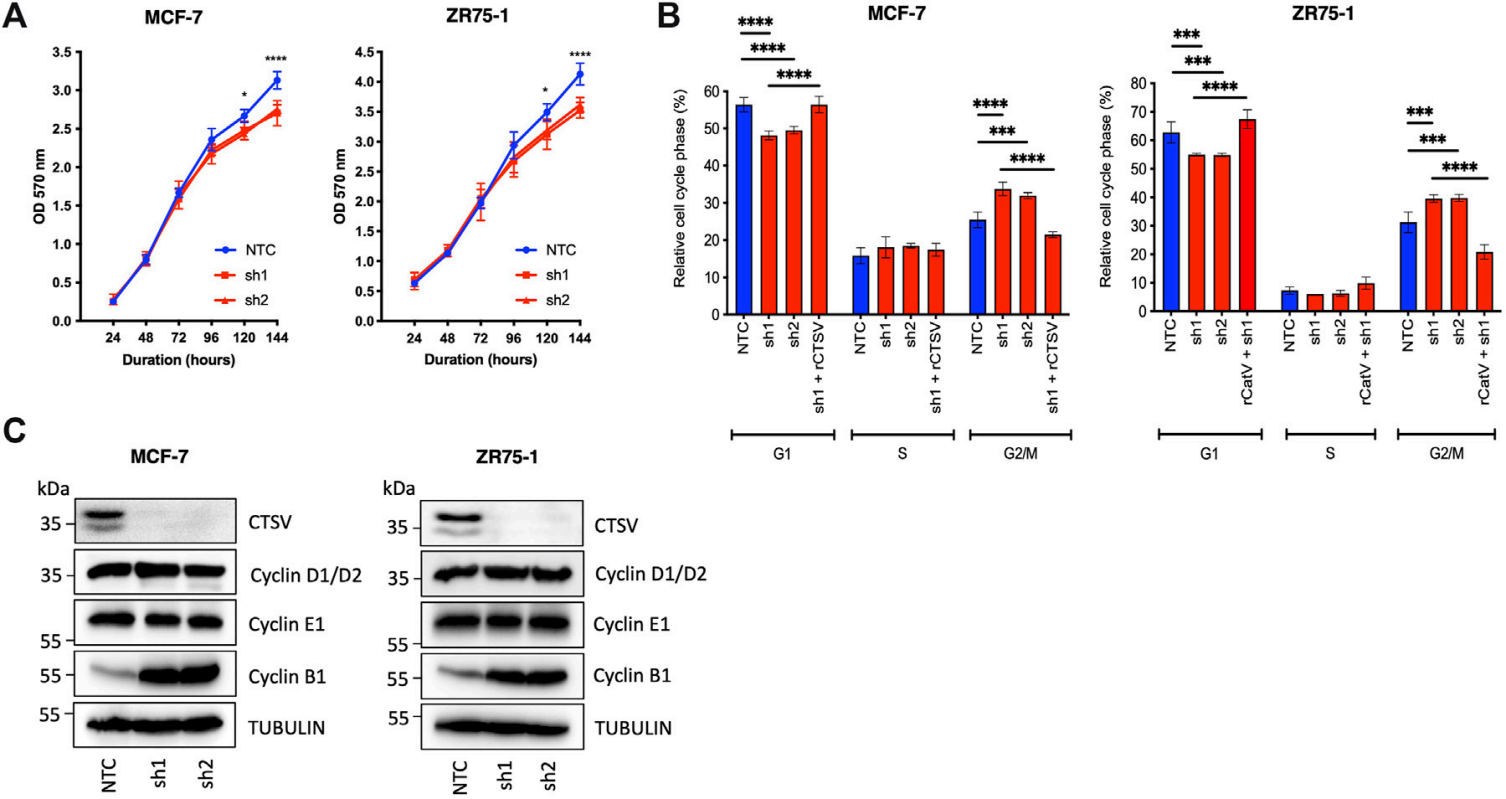
CTSV depletion attenuates growth of breast cancer cells by stalling progression through G2/M phase. **(A)** CTSV depletion in MCF-7 and ZR75-1 cells results in reduced cell growth when assessed by MTT assay. **(B)** PI staining with flow cytometry analysis identified that CTSV depletion results in fewer cells in G1, with a concomitant increase in G2/M phase. Rescue experiments were undertaken by restoring CTSV expression in sh1 cells, where cell cycle profiles were restored to that of the NTC cells. **(C)** Western blotting analysis of CTSV depleted cells shows that cyclin B1 expression is elevated in comparison to control cells, whereas cyclins D1/D2 and E1 remain unchanged. Tubulin expression was used as an internal loading control, with presented blots representative of at least three independent experiments. The average and standard deviation (SD) values are representative of three independent experiments, with statistical analysis was determined by two-way-ANOVA with a Tukey’s *post hoc* test for multiple comparisons performed using GraphPad Prism 8.

Previous studies have suggested that CTSL is required for cell cycle progression of colorectal cancer cells (Tamhane et al., 2016) and glioma cells (Zhang et al., 2015), with a similar observation made in our examination of CTSV in thyroid carcinoma cells (Al-Hashimi et al., 2020a). Therefore, to determine whether CTSV may have a similar role in breast cancer cells, we examined the impact of CTSV on cell proliferation using the MCF-7 and ZR75-1 shRNA cell line models, as characterised and reported in our previous publication (Sereesongsaeng et al., 2020). MCF-7 and ZR75-1 cells are both ER+ breast cancer cell lines that have been classified as Luminal A and have been extensively used to study this particular subtype of the disease (Dai et al., 2017). Neither depletion or overexpression of CTSV had any impact on CTSL protein levels within the cells (Supplementary Figure S7). Examination of these cells identified that CTSV depletion resulted in a significant reduction in cell growth, but this was only evident >120 h after seeding (Figure 1A). In order to ascertain if this was mediated by altering cell cycle progression, propidium iodide staining of non-synchronised cells was undertaken to determine the relative proportion of cells within each stage of the cell cycle. Flow cytometry analysis showed that the CTSV depleted cells (sh1 and sh2) had significantly fewer cells in G1 phase, compared to the NTC control cells. Conversely, when G2/M phase was examined, a greater proportion of the CTSV depleted cells presented in this phase compared to the NTC control cells. Re-expression of CTSV by transfection of an shRNA resistant CTSV construct (rCTSV) into sh1 cells restored the proportion of cells within both G1 and G2/M phases of the cell cycle to that observed in the NTC control cells, suggesting that CTSV depletion was the key mediator for changes to the cell cycle profile (Figure 1B). To corroborate these results, Western blotting analysis for cyclin D1/D2, E1 and B1 expression was undertaken. In support of the cell cycle analysis by flow cytometry, cyclin B1 expression was markedly elevated in the CTSV depleted cells, whereas all other cyclins were unchanged, suggesting that the CTSV depleted cells progress more slowly through the G2/M phase of the cell cycle (Figure 1C).

### Histone H3 and H4 expression is regulated by CTSV

**FIGURE 2:**
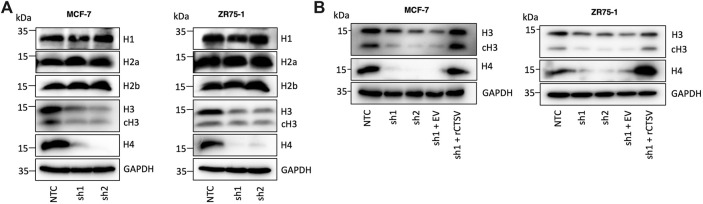
Histone H3 and H4 protein expression is regulated by CTSV. (**A)** Western blotting analysis of histone protein expression shows a reduction in histone H3 and H4 in CTSV depleted cells, with no impact on histones H1, H2a and H2b. **(B)** Confirmation of CTSV impact on histone H3 and H4 was ascertained using rescue experiments and the re-expression of CTSV in shCTSV-1 cells. GAPDH expression was used as an internal loading control and data presented is representative of at least three independent experiments.

Having determined the impact of CTSV depletion on cell cycle progression, our aim was to determine how these effects could be mediated. Several studies have previously identified that cathepsins can regulate histone proteins by proteolytic cleavage (Duncan et al., 2008; Adams-Cioaba et al., 2011; Daura et al., 2021). To determine if CTSV may elicit a similar role in breast cancer cells, we examined expression of 4 core histone proteins (H2a, H2b, H3 and H4) as well as linker protein histone H1. Western blotting analysis using the MCF-7 and ZR75-1 shCTSV models revealed no impact on histone H1, H2a or H2b, however a notable reduction in H3 and H4 expression was evident in the CTSV depleted cells (Figure 2A). To confirm the impact of CTSV on histone H3 and H4 expression, restoration of CTSV expression by transfection of rCTSV into the sh1 cells was undertaken. Western blotting analysis illustrated that restoration of CTSV expression could reinstate histone H3 and H4 expression levels to that observed in the control cells, confirming the involvement of CTSV in the process (Figure 2B). RQ-PCR analysis of H3 and H4 expression revealed that CTSV did not transcriptionally regulate their expression, suggesting that CTSV must exert its effects at the protein level (Supplementary Figure S8).

Numerous publications have identified that H3 is subjected to proteolytic processing at numerous sites within the histone tail, with reports that these cleavages are important for epigenetic regulation, cell differentiation and gene expression (Santos-Rosa et al., 2009; Zhou et al., 2014). While the Western blotting analysis in Figure 2A identified reduced expression of full-length histone H3, diminished expression of the cleaved form was also evident. Therefore, to determine whether the diminished H3 expression was due to CTSV cleavage of H3, *in vitro* cleavage assays were undertaken, incubating recombinant active CTSV with recombinant H3. No cleavage of H3 was evident at any timepoint examined. As a control, recombinant H3 was incubated with active human CTSL, where full processing of H3 was evident within 2 h, in agreement with previous reports (Supplementary Figure S9) (Duncan et al., 2008; Adams-Cioaba et al., 2011). This intriguing observation then led us to question whether CTSV could possibly have a role in stabilising histone H3 and H4 protein expression.

### CTSV mediates histone H3 and H4 stability by regulation of histone chaperone sNASP

**FIGURE 3:**
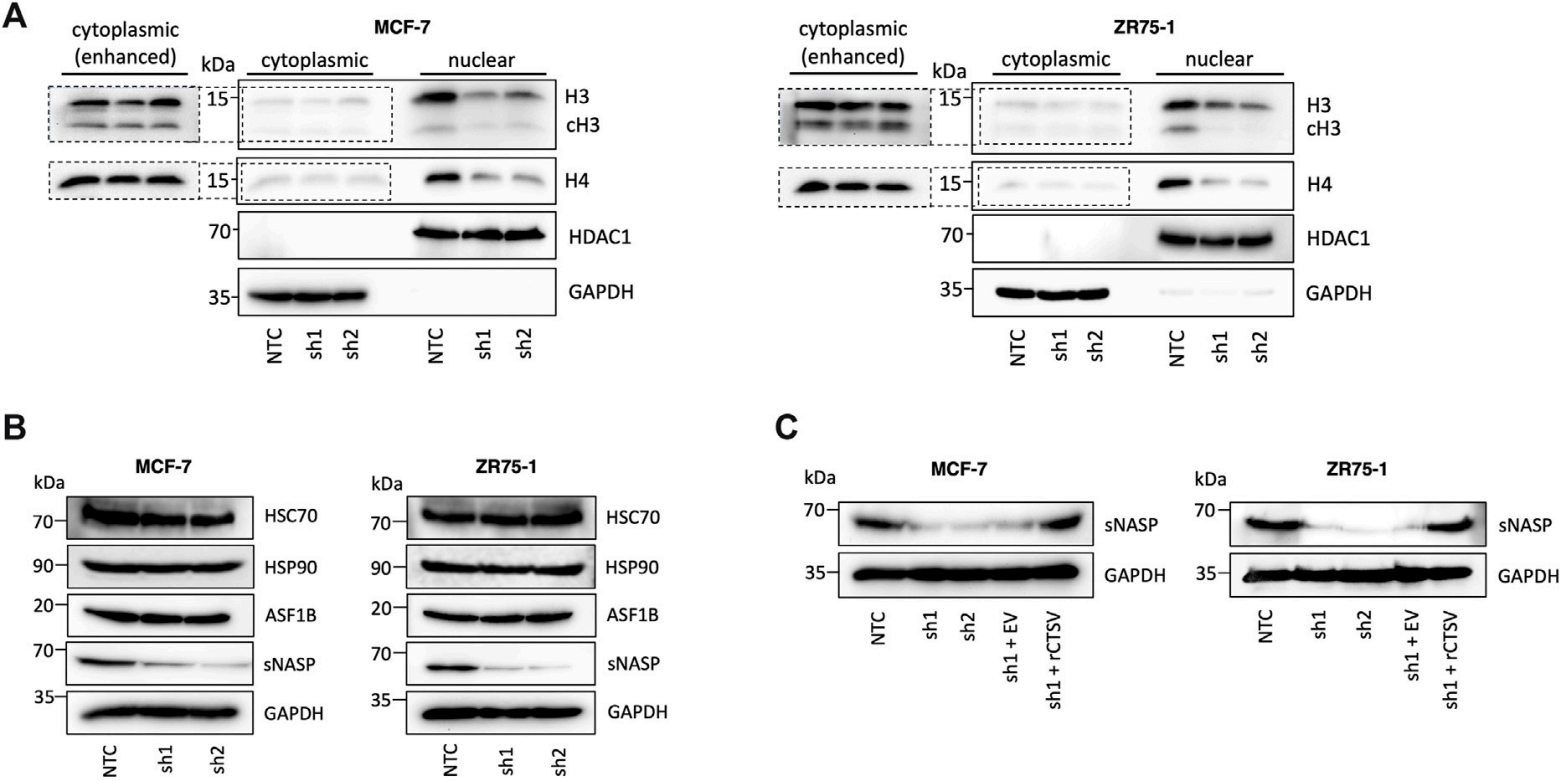
CTSV mediates histone H3 and H4 nuclear stability via the regulation of chaperone sNASP. (**A)** Western blotting analysis shows that CTSV depletion results in a reduction in histone H3 and H4 protein levels in the nucleus of MCF-7 and ZR75-1 cells. Enhanced exposure of cytoplasmic fractions shows that there is no apparent change in histone H3 or H4 expression levels in this compartment of the cells. **(B)** Examination of the key histone H3 and H4 chaperones HSC70, HSP90, ASF1B and sNASP by Western blotting identified that sNASP protein levels are lower in CTSV depleted cells, while all other chaperones remain unchanged. **(C)** Confirmation of the involvement of CTSV on sNASP protein expression undertaken by rescue experiments, restoring CTSV expression in sh1 cells, which restores sNASP levels to that of the control cells. HDAC1 and GAPDH expression were used as loading controls relative to the nuclear and cytoplasmic fractions respectively and data presented is representative of at least three independent experiments.

The role of CTSV in regulating histone H3 and H4 was further evaluated by assessing their relative expression in cytoplasmic and nuclear fractions from the MCF-7 and ZR75-1 cells. As expected, histone protein expression was predominantly found within the nuclear fractions, and it was very apparent that the CTSV depleted cells displayed reduced histone H3 and H4 protein expression within the nucleus compared to control cells (Figure 3A). While total histone H3 and H4 protein expression is notably lower in cytoplasmic fractions, examination under enhanced exposure and subsequent densitometry analysis, suggested that CTSV had no impact on cytoplasmic histone H3 or H4 levels (Figure 3A; Supplementary Figure S10).

As histone H3 and H4 expression was only altered by CTSV depletion in the nuclear fractions, the lack of accumulation in the cytoplasm suggested that these effects may not be a result of impairments of histone trafficking from the cytoplasm where they are synthesised, but instead may arise from changes to histone stability once trafficked to the nucleus. The regulation of histone expression is crucial for cell homeostasis and cell cycle progression, with histone stability mediated by several chaperone proteins, including Asf1, sNASP, Hsc70 and Hsp90 (Cook et al., 2011). To determine the impact of CTSV on histone H3 and H4 stability, Western blotting analysis of these chaperones in the CTSV-depleted cells was undertaken. The results revealed that only sNASP expression was altered by CTSV expression, with a notable reduction in sNASP expression in the cells lacking CTSV (Figure 3B). Reintroduction of CTSV expression, again using rCTSV, restored sNASP expression to control condition levels, suggesting that CTSV has a role in regulating sNASP expression (Figure 3C). RQ-PCR analysis of sNASP expression in each of the cell line models shows no reduction in sNASP mRNA levels following CTSV depletion, suggesting that the impact of CTSV on sNASP is not transcriptionally mediated (Supplementary Figure S11).

### CTSV is localised to the nuclear compartment of breast cancer cells

**FIGURE 4:**
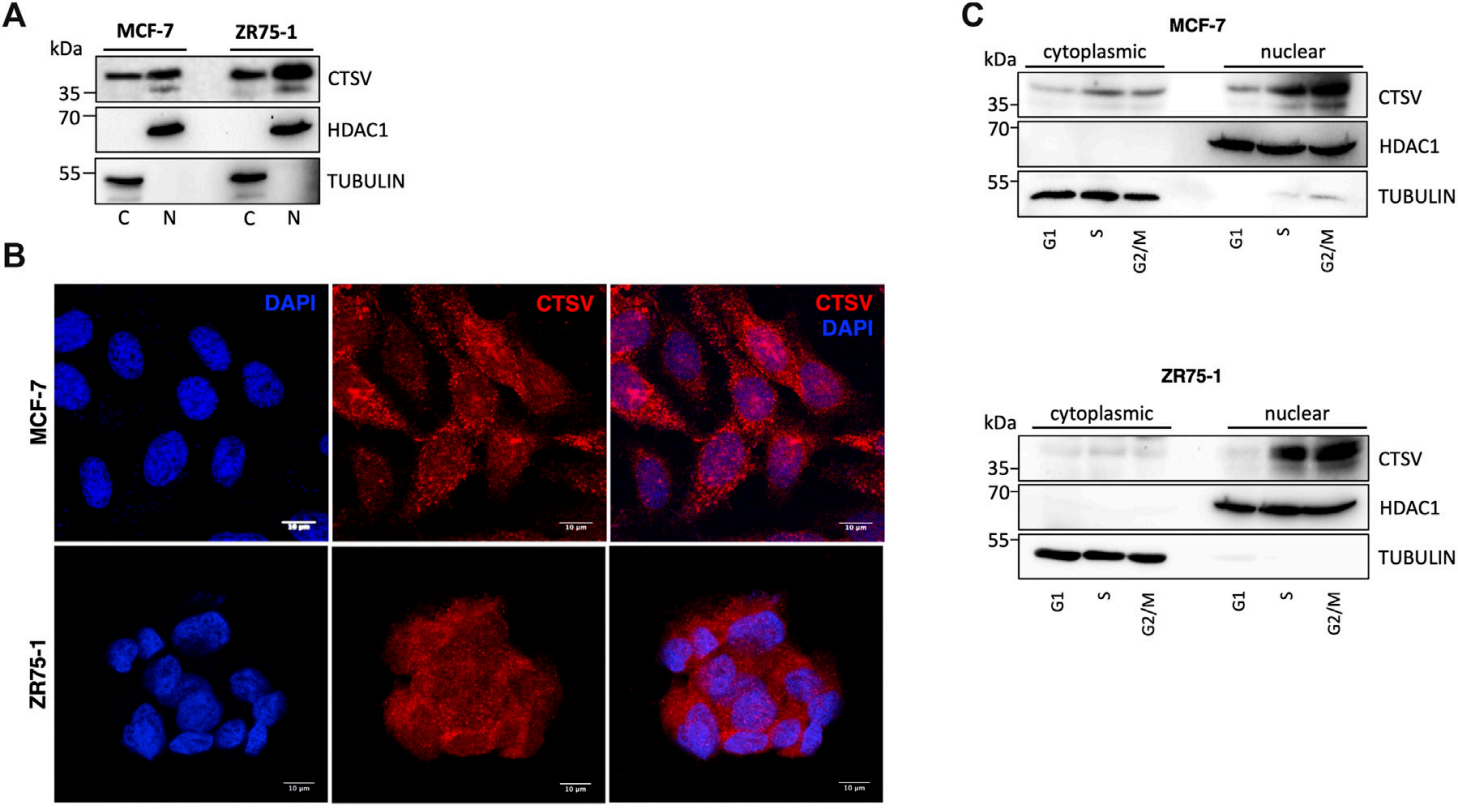
CTSV is localized in the nuclear compartment of breast cancer cells. **(A)** Cytoplasmic (C) and nuclear (N) extractions from MCF-7 and ZR75-1 cells identified the presence of CTSV in both cellular compartments by Western blotting. **(B)** Confocal microscopy was undertaken to confirm the nuclear localisation of CTSV (red) with DAPI nuclear staining (blue). Scale bars = 10 μm **(C)** Cells sorting and Western blotting analysis suggested that CTSV is localised to the nucleus predominantly during S and G2/M phases. HDAC1 and tubulin expression were included as loading controls relative to the nuclear and cytoplasmic fractions respectively and data presented is representative of at least three independent experiments.

We then examined the localisation of CTSV in the breast cancer cells in order to understand the relationship between CTSV, histone H3/H4 and sNASP in more detail. Sub-cellular fractionation and Western blotting identified that CTSV protein expression was evident in both cytoplasmic C) and nuclear N) fractions from the MCF-7 and ZR75-1 cells. Control blots using HDAC1 as a nuclear marker and tubulin as a cytoplasmic marker confirmed purity of the protein lysates (Figure 4A). To verify CTSV nuclear localisation, confocal microscopy was performed using an anti-CTSV antibody (red) and a DAPI nuclear stain (blue). CTSV expression was evident as punctate lysosomal staining as well as expression within the nuclear compartment, corroborating the Western blotting data (Figure 4B).

Previous studies in thyroid carcinoma cells suggested that CTSV trafficking to the nucleus was most abundant during S phase of the cell cycle (Al-Hashimi et al., 2020a). Therefore, to evaluate whether this is the case for CTSV in the breast tumour cells, cell synchronisation was undertaken using thymidine and nocodazole, prior to nuclear extraction. Western blotting analysis clearly shows that CTSV is predominantly localised to the nucleus during S phase, with the protein maintaining this sub-cellular localisation during G2/M phase (Figure 4C).

### Nuclear CTSV trafficking is mediated by NLS and importins

**FIGURE 5:**
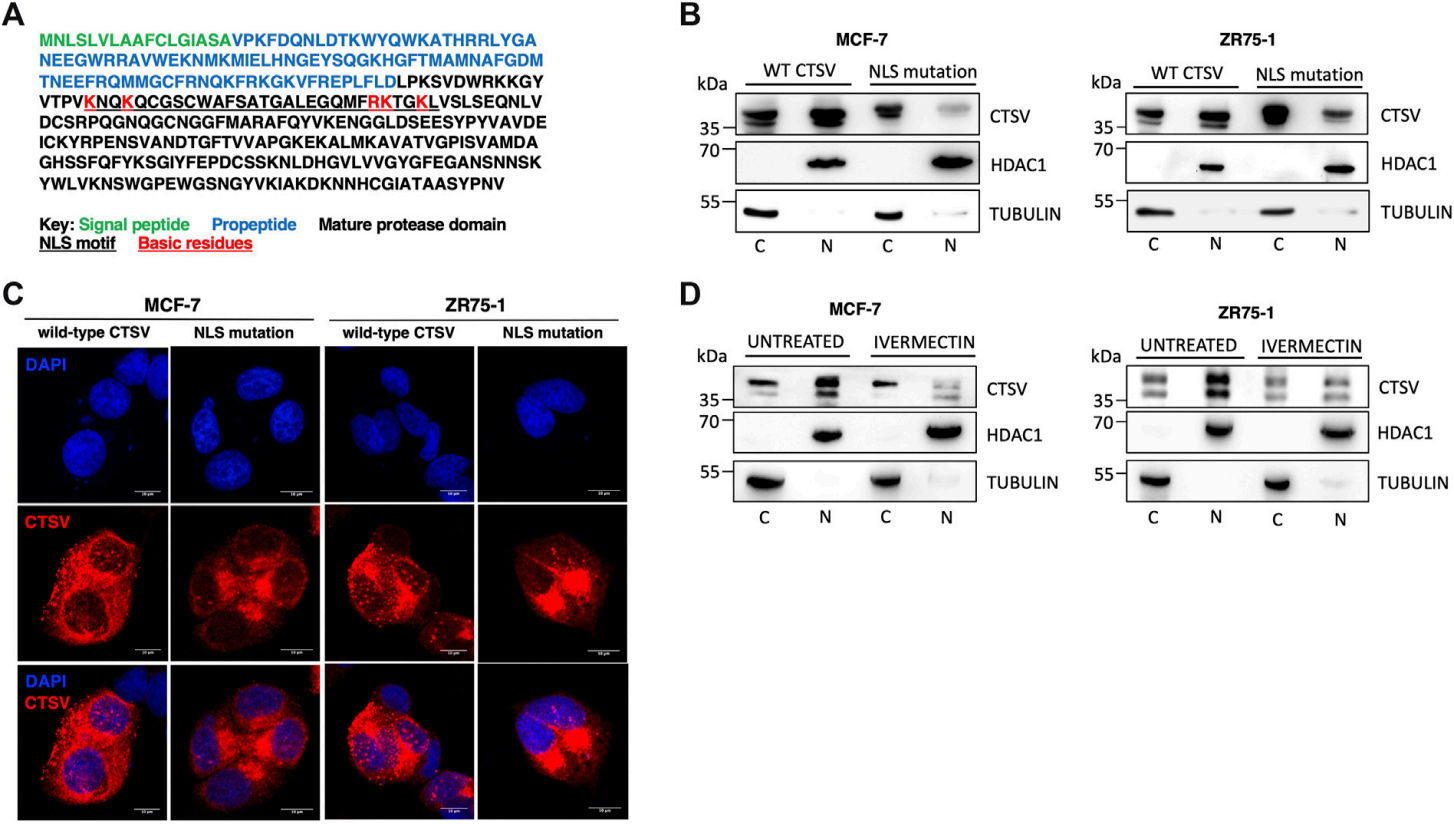
Nuclear CTSV trafficking is mediated via a bipartite nuclear localisation signal. **(A)** *In silico* analysis via NLS mapper has identified a potential bipartite NLS sequence motif (underlined residues) within CTSV. A CTSV expression construct with a mutated NLS was generated (mtNLS) by mutation of the basic lysine and arginine residues within the NLS motif to alanines (indicated in red). **(B)** Western blotting analysis following transfection of the mtNLS expression construct shows reduced nuclear CTSV protein expression in MCF-7 and ZR75-1 cells. **(C)** Confocal analysis of CTSV protein expression (red) following transfection of the mtNLS construct was used to confirm reduced nuclear CTSV expression. DAPI counterstaining (blue) was used to identify cell nuclei. Scale bars = 10 μm **(D)** MCF-7 and ZR75-1 cells were treated with ivermectin to confirm the functionality of the predicted NLS motif. Western blotting analysis of cytoplasmic (C) and nuclear (N) fractions confirmed nuclear localisation of CTSV was reduced following ivermectin treatment. HDAC1 and tubulin expression were included as loading controls relative to the nuclear and cytoplasmic fractions respectively and data presented is representative of three independent experiments.

Having confirmed that CTSV was localised to the nuclear compartment, the question arose as to how this would occur. *In silico* analysis of the CTSV protein sequence using the online nuclear localisation signal (NLS) prediction tool, NLS mapper (http://nls-mapper.iab.keio.ac.jp) (Kosugi et al., 2009), suggested that CTSV contains a potential bipartite nuclear localisation signal between residues lysine 130 and lysine 159 (underlined residues) (Figure 5A). To examine the functionality of this predicted sequence, mutagenesis of 5 key basic residues (lysine and arginine residues, highlighted in red) within the NLS sequence was undertaken, with conversion to alanine residues as reported in similar studies (Theodore et al., 2008; Liu et al., 2010; Pawlowski et al., 2010). Confirmation that the resultant NLS mutant construct (mtNLS) retains CTSV functionality was confirmed by transfecting cells with both wtCTSV and mtNLS and blotting for GATA3, in line with our recently published results where overexpression of active CTSV facilitates GATA3 protein turnover (Sereesongsaeng et al., 2020) (Supplementary Figure S12). Transfection of the CTSV mtNLS construct revealed distinct differences in the CTSV expression pattern compared to the wtCTSV transfected cells. The mtNLS expressing cells exhibited a notable reduction in the levels of nuclear CTSV protein expression, evident in both the MCF-7 and ZR75-1 cells by Western blotting (Figure 5B). Subsequent confocal microscopy analysis was also undertaken, confirming that transfection of mtNLS led to a notable reduction in CTSV within the nuclear compartment (Figure 5C).

Extensive research has shown that the importins are required for the nuclear trafficking of proteins containing NLS sequences (Lu et al., 2021). To further assess the NLS sequence identified within CTSV, cells were treated with the importin inhibitor ivermectin, to confirm that blockade of the classical nuclear import pathway mediated by importin-α and importin-β1 was involved in the nuclear trafficking of CTSV. To ensure any observed effects were not due to cytotoxicity associated with the generic impairment of nuclear trafficking, ivermectin concentrations and timepoints were optimised to exclude significant impact on MCF-7 and ZR75-1 cell viability (Supplementary Figure S13). After treatment with ivermectin (10 μM for 24 h), endogenous CTSV expression was evaluated by nuclear and cytoplasmic extraction. In agreement with previous results, CTSV nuclear expression was notably reduced following treatment with ivermectin in both the MCF-7 and ZR75-1 cells, suggesting that the identified NLS motif and importin-mediated trafficking are a viable mechanism for CTSV localisation to the nucleus of breast cancer cells (Figure 5D).

### Nuclear CTSV regulates cell cycle progression in breast cancer cells

**FIGURE 6:**
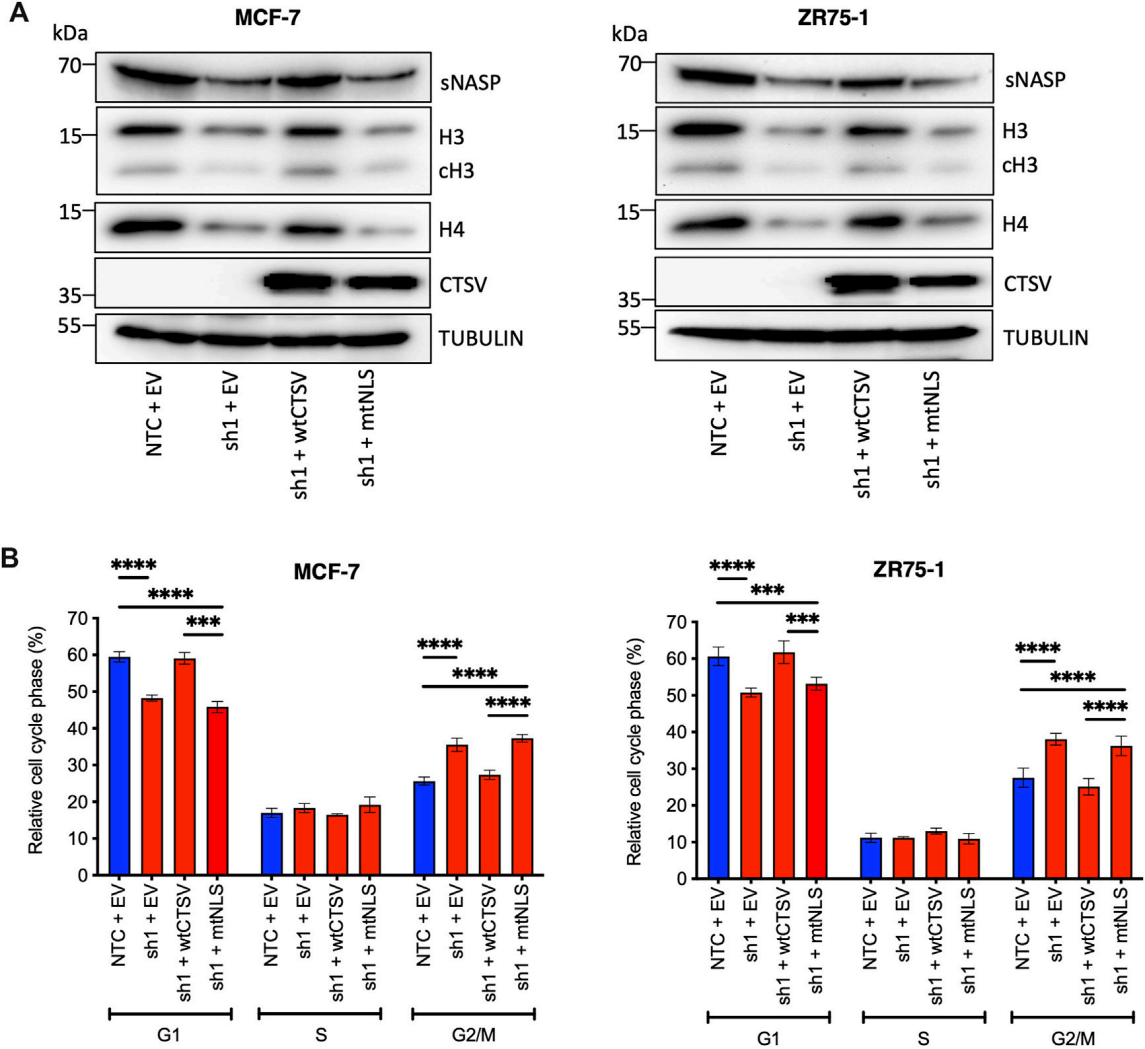
Nuclear CTSV is required for histone H3, H4 and sNASP protein expression and cell cycle progression. **(A)** Restoration of wtCTSV (which traffics to the nucleus) expression in CTSV depleted cells (sh1) is necessary to reinstate histone H3, H4 and sNASP protein expression, while restoration from the mtNLS construct (which has impaired nuclear trafficking) is insufficient. **(B)** Confirmation of the impact of nuclear CTSV on cell cycle progression was observed by PI staining and flow cytometry. Restoration of CTSV from the wtCTSV construct was necessary to restore G1 and G2/M cell proportions to that observed in the NTC cells, restoration of CTSV from the nuclear deficient mtNLS construct had no impact on the cell cycle profile. Tubulin expression was used as an internal loading control, with presented blots representative of three independent experiments. The average and standard deviation (SD) values are representative of three independent experiments, with statistical analysis was determined by two-way-ANOVA with a Tukey’s *post hoc* test for multiple comparisons performed using GraphPad Prism 8.

To ascertain whether the impact on sNASP and histone stability was mediated by nuclear CTSV, CTSV expression was restored in the CTSV-depleted cells using the wild-type CTSV (wtCTSV) and NLS mutant CTSV constructs (mtNLS). Our earlier results confirmed that while wtCTSV transfection resulted in cytoplasmic and nuclear expression of CTSV, mtNLS was largely excluded from the nuclear compartment (Figure 5B). As such these constructs were expressed in the CTSV-depleted cells as tools to examine the relative contribution of nuclear CTSV. Restoration of wtCTSV expression in both the MCF-7 and ZR75-1 shCTSV-1 cells restored sNASP, H3 and H4 expression levels to those observed in the NTC control cells, whereas expression of mtNLS CTSV has no discernible effect (Figure 6A, Supplementary Figure S14). Furthermore, assessing the impact of CTSV restoration on cell cycle progression, identified that expression of wtCTSV in the CTSV-depleted cells restored cell cycle progression to that observed in the NTC cells, whereas the cells transfected with mtNLS CTSV, which has diminished trafficking to the nucleus, remain impaired (Figure 6B). Collectively these results suggest that nuclear CTSV is required for histone stability and cell cycle progression in breast cancer cell lines, through regulating the protein expression of the chaperone protein sNASP.

## Discussion

While much of the early work focused on the role of the cysteine cathepsins within the endo-lysosomal compartment, increasing evidence now supports the functionality of these proteases in alternative locations such as the extracellular environment, the mitochondria and, in particular, the nucleus (Goulet et al., 2004; Müntener et al., 2004; Goulet et al., 2007; Duncan et al., 2008; Maubach et al., 2008; Tedelind et al., 2010; Duarte et al., 2014; Tamhane et al., 2016; Soond et al., 2019; Vidak et al., 2019; Al-Hashimi et al., 2020a). We recently reported the presence of CTSV in the nucleus of thyroid carcinoma cells (Al-Hashimi et al., 2020a) and the data presented in this study has now identified that CTSV can translocate to the nuclear compartment of breast carcinoma cells as well.

Since the identification of nuclear cathepsins, questions have arisen as to how cysteine cathepsin species could ultimately reside within the nucleus and what their purpose there might be. Several studies have identified or proposed truncated variants, most of which lack a portion of their N-terminal protein sequence (Mehtani et al., 1998; Goulet et al., 2004; Sansanwal et al., 2010; Tedelind et al., 2010; Al-Hashimi et al., 2020a). Research suggested the truncated nuclear protease species were due to alternative splicing or initiation of transcription from downstream AUG sites, while work from the Reinheckel lab suggested that this was not the case, at least for CTSL (Tholen et al., 2014). Within this research, the predominant species detected in the nuclear compartment of the breast cancer cell lines was the proform of the protease, with no evidence of a truncated species. Preliminary overexpression experiments suggest that the mature form of CTSV is present within the nuclear compartment (data not shown), however capturing the endogenous mature form has proven challenging due to low expression levels. More in-depth experimentation is needed to confirm the presence of mature CTSV within the nucleus and determine whether this is a result of proCTSV activation or whether it can be trafficked to the nuclear compartment itself—the presence of the NLS motif on the mature protease domain suggests this could be feasible.

While previous reports suggested that CTSV did not contain an NLS sequence (Tedelind et al., 2010), updated knowledge including increased length of the linker region between the two basic residue clusters of bipartite domains, has refined how software can predict potential NLS motifs. Mutagenesis of lysine and arginine residues within the predicted NLS to alanine residues and treatment with importin inhibitor, ivermectin, implies that CTSV does contain a functional bipartite NLS sequence. To the best of our knowledge, this is the first confirmation of an NLS motif present within a cysteine cathepsin. Sullivan and others previously suggested the presence of an NLS-like sequence within CTSL but there has been no examination of its functionality (Sullivan et al., 2009). Likewise, studies examining artificially truncated CTSB variants reported the lack of a canonical NLS motif but predicted a signal patch within the heavy chain of the protease was responsible for nuclear targeting (Bestvater et al., 2005). Other research has implicated nuclear trafficking of the cathepsins to be facilitated via complex formation with other NLS-containing proteins. CTSL has been shown to traffic to the nucleus via the transcription factor Snail, which contains an NLS sequence. Mutation of the lysine and arginine residues within the Snail NLS, led to an accumulation of CTSL in the cytoplasm, whilst importin β-1 knockdown further reduced the nuclear localisation of CTSL (Burton et al., 2017a).

The identification of non-canonical localization of CTSV to cellular compartments such as the nucleus raises pertinent questions as to the extent of the biochemical roles of proteases within the cell. In this research, we have identified that CTSV has an important role in the stabilisation of histones H3, H4 and their chaperone sNASP, which appears to be important for cell cycle progression. Numerous studies have implicated that the cathepsins have specific roles within the nuclear compartment including the regulation of cell proliferation, transcription factor and histone processing (Goulet et al., 2004; Goulet et al., 2007; Duncan et al., 2008; Khalkhali-Ellis et al., 2014; Bach et al., 2015; Tamhane et al., 2016; Al-Hashimi et al., 2020b). Previous research has also documented interactions between the histones and cathepsins, beyond proteolytic cleavage. Čeru and others identified that the inhibition of CTSL by stefin B was potentiated in the presence of histone H3, suggesting that protease inhibitors in the nucleus could regulate protease activity within the nucleus, potentially protecting substrates from processing (Čeru et al., 2010). Similar observations were made when the presence of DNA was found to accelerate the inhibition of CTSV by the nuclear serpin MENT (Ong et al., 2007).

Examination of CTSV protein expression in the context of the cell cycle, identified that the protease appears to translocate to the nucleus during S phase, remaining there during G2/M. This complements our recent work in thyroid carcinomas which suggested that nuclear CSTV peaks during S phase (Al-Hashimi et al., 2020a). Furthermore, depletion of CTSV by shRNA results in impaired proliferation, with an accumulation of cells within the G2/M phase of the cell cycle, complemented by increased cyclin B1 expression, suggesting that the cells exhibit delayed progression through this phase. While the phases of G2 and M were not discriminated in this study, previous work has reported that cyclin B1 levels are significantly elevated prior to cells entering mitosis, with rapid degradation as cells proceed through anaphase (Widrow et al., 1997). While CTSV depletion results in slower cell cycle progression, it does not appear to induce cell cycle arrest or lead to apoptosis, as was evident from the lack of an increasing sub-G1 population. Previous studies also support our findings with research implicating that nuclear CTSD and CTSL can also impact on cell cycle progression in breast cancer and colorectal cancer cell lines respectively (Bach et al., 2015; Tamhane et al., 2016). Whether any redundancy or compensation between the cathepsins exists in the nuclear compartment, remains to be determined. Previous work by Burton and others illustrated that CTSL was not present in the nuclear compartment of the MCF-7 cells, and it was only following overexpression of the transcription factor Snail, resulting in the promotion of EMT, that nuclear CTSL became evident (Burton et al., 2017b). However, research examining colorectal cancer has identified both CTSL and CTSV in the nuclear compartment (Tamhane et al., 2016), therefore it is plausible that redundancy may exist in certain models.

The association between the cathepsins and histones has been reported on several occasions, with most reports focusing on cathepsin proteolytic processing of histones (Duncan et al., 2008; Adams-Cioaba et al., 2011). Within this study, the data suggests that CTSV regulates histones, not by mediating their proteolytic cleavage, but by regulating expression of the histone H3 and H4 chaperone, sNASP. As histones are crucial components for DNA replication during S phase, any impairment in the histone supply to the chromatin can prolong cell cycle progression (Nelson et al., 2002; Günesdogan et al., 2014).

sNASP has an affinity to bind to the core histone (histone H3-H4 complexes) and the linker histone (histone H1) which regulates the sub-cellular dynamic of histones and maintains the stability of histone H3 and H4 (Finn et al., 2008; Wang et al., 2008). Depletion of sNASP significantly reduced the levels of histone H3 and H4, by directing them for degradation by chaperone-mediated autophagy. Research has shown that silencing sNASP in different cell lines (Hela, 293T, and U-2 OS cells) significantly reduced expression of histone H3 and H4 only, with no effect on other histones. Additionally, depletion of sNASP did not change histone H3 and H4 mRNA level, as observed here, suggesting that the depletion of sNASP specifically affects the histone H3 and H4 pool without affecting their transcription (Cook et al., 2011).

While there have been extensive efforts in the development of inhibitors that selectively target other cathepsin family members, only a small number of CTSV-selective inhibitors have been developed to date. Previous work by Marques et al., detailed the evaluation of acridones and 4-quinolines as CTSV inhibitors, however many also had the ability to target CTSL. Interestingly, some *N*-arylanthranilic acid-based compounds displayed some selectivity for CTSV over CTSL, which may be an avenue worth exploring more in future (Marques et al., 2012). More recently, a virtual high-throughput strategy was used to screen commercial compound libraries to identify new inhibitors targeting CTSV. While some of the most potent CTSV inhibitors identified could also target CTSL, compound 7 from the study did show selectivity towards CTSV and examination in cell-based assays illustrated its ability to attenuate MCF-7 tumour cell proliferation, in line with our observations herein using shRNAs (Mitrović et al., 2022).

Whether any of these CTSV-targeting compounds has the ability to enter the nucleus and inhibit the protease in this compartment is currently unknown. Therefore, to ascertain if the impact of CTSV was mediated by the nuclear species, the NLS-mutant CTSV construct, which was proven to retain catalytic activity but lacked the ability to traffic to the nucleus, was used to determine whether CTSV in the nucleus played a role in the regulation of sNASP. The results reveal that the level of sNASP, as well as histone H3 and H4, could not be restored in the CTSV depleted cells when CTSV cannot enter into the nucleus. In addition, the cell cycle analysis illustrated that the re-expression of CTSV in the depleted cells can restore the cell cycle to normal. This finding confirms that CTSV in the nucleus has an important role to regulate sNASP expression.

## Conclusion

In conclusion, this research demonstrates that CTSV plays an important role in the regulation of histone H3 and H4 protein expression via regulation of their chaperone, sNASP, however further research is needed to ascertain the molecular mechanism by which CTSV regulates sNASP. Depletion of CTSV results in slowed progression though the cell cycle, which appears to be mediated by the protease when localised in the nucleus. Targeting CTSV in tumours such as breast cancers may be of interest as a therapeutic strategy to impair breast cancer cell progression. However, further examination of the paradigm changing localisation of CTSV is critical to understanding the tumour cell intrinsic role of CTSV in breast cancer, which could be exploited for future development of novel therapeutics.

## Data Availability

The raw data supporting the conclusion of this article will be made available by the authors, without undue reservation.

## Supplementary material

SUPPLEMENTARY FIGURES S1 to S6 Uncropped images of western blotting results.

SUPPLEMENTARY FIGURE S7 Alteration to CTSV expression has no impact on CTSL protein levels. a CTSV depletion by shRNA or b CTSV overexpression had no impact on CTSL protein expression within the MCF-7 cells. Tubulin was used as an internal loading control and data presented is representative of two independent experiments.

SUPPLEMENTARY FIGURE S8 CTSV does not transcriptionally regulate histone H3 and H4 expression. RQ-PCR was used to examine mRNA expression of histone H3 and H4 in NTC and shCTSV cell lines (sh1 and sh2) from MCF-7 and ZR75-1 cell line models. Data is presented as the average and standard deviation (SD) from three independent experiments, with mRNA expression presented relative to the NTC control cells.

SUPPLEMENTARY FIGURE S9 CTSV does not cleave histone H3. In vitro cleavage assay with recombinant human H3 protein (rhH3) incubated with activated CTSV or CTSL recombinant proteins for the indicated timepoints. Proteins were resolved by SDS-PAGE and visualised by GelCode™ Blue staining. No cleavage of rhH3 is evident following incubation with CTSV, whereas full processing to the cleaved H3 fragment is evident following incubation with CTSL. Data presented is representative of three independent experiments.

SUPPLEMENTARY FIGURE S10 Densitometry of histone H3, cleaved H3 and H4 protein expression in NTC and shCTSV cells. Densitometry analysis confirms that CTSV depletion has no effect on cytoplasmic expression of histone H3, cleaved H3 or H4, whereas nuclear expression is dramatically impaired. Similar effects are noted in both MCF-7 and ZR75-1 cell line models. Data is presented as the average and standard deviation (SD) relative densitometry units collated from three independent experiments.

SUPPLEMENTARY FIGURE S11 CTSV does not transcriptionally regulate sNASP expression. RQ-PCR was used to examine mRNA expression of sNASP in NTC and shCTSV cell lines (sh1 and sh2) from MCF-7 and ZR75-1 cell line models. Data is presented as the average and standard deviation (SD) from three independent experiments, with mRNA expression presented relative to the NTC control cells.

SUPPLEMENTARY FIGURE S12 CTSV mtNLS construct expresses functional CTSV. To confirm that the CTSV mtNLS construct produces a functional CTSV protein, MCF-7 and ZR75-1 cells were transfected with wtCTSV, mtNLS or empty vector (EV) control for 48 h. GATA3 protein expression was examined by western blotting, with reduced GATA3 protein expression evident following wtCTSV and mtNLS expression. Tubulin was used as an internal loading control and data presented is representative of three independent experiments.

SUPPLEMENTARY FIGURE S13 Cell viability assay for ivermectin treatment. MTT assay was used to confirm that the ivermectin concentration (10 μM) and timepoint (24h) used had no significant impact on MCF-7 or ZR75-1 cell viability. Data is representative of three independent experiments, with the percentage of cell viability and standard deviation (SD) presented relative to the untreated control.

SUPPLEMENTARY FIGURE S14 Densitometry of histone H3, cleaved H3, H4 and sNASP protein expression following restoration of CTSV expression from wtCTSV and mtNLS constructs. Densitometry analysis confirms that CTSV restoration from wtCTSV is required to reinstate histone H3, cH3, H4 and sNASP protein expression, while restoration from the mtNLS construct (which has impaired nuclear trafficking) is insufficient. Similar effects are noted in both MCF-7 and ZR75-1 cell line models. Data is presented as the average and standard deviation (SD) relative densitometry units collated from three independent experiments.
